# Pharmacological and Medicinal Properties of the South American Medicinal Plant *Bowdichia virgilioides* Kunth and Its Bioactive Products

**DOI:** 10.3390/life16020358

**Published:** 2026-02-20

**Authors:** Christian Bailly

**Affiliations:** 1 UMR9020 CNRS—U1366 Inserm, CHU de Lille, Université de Lille, 59000 Lille, France; christian.bailly@univ-lille.fr; 2 Institute of Pharmaceutical Chemistry Albert Lespagnol (ICPAL), Faculty of Pharmacy, University of Lille, 59006 Lille, France; 3 OncoWitan, 59290 Lille, France

**Keywords:** *Bowdichia virgilioides*, sucupira seeds, phytochemicals

## Abstract

*Bowdichia virgilioides* Kunth is a tree largely present in South America, notably in the Cerrado savannah. The species is known for the quality of its dense and resistant wood, used in construction and furnishing. *B. virgilioides* is also a medicinal plant used, from leaves to roots, for the treatment of various human pathologies (pharyngitis, bronchitis, healing wounds, diabetes, and arthritis). The present review provides an analysis of the scientific literature pertaining to *B. virgilioides*, with a focus on pharmacological activities. Aqueous and organic extracts have been used to treat inflammatory pathologies and to combat infectious diseases caused by microorganisms and parasites. All phytochemicals at the origin of the bioactivities of extracts have been identified, including 37 terpenoids, 8 alkaloids, 21 flavonoids and 13 other products. All natural products are discussed, with a focus on a dozen compounds with well-documented pharmacological properties and/or a known mechanism of action. Key products include ormosanine (alkaloid), vouacapane (diterpenoid), lupeol (terpenoid), isoquercetin (flavonoid), isocordoin (chalcone), and little-known specific products (bowdichine and bowdenol). The botanical and phytochemical analysis shed light on this valuable Fabaceae species with the objective to promote its preservation and cultivation, as well as further pharmacological investigations aimed at rationalizing its long-established ethnobotanical use.

## 1. Introduction

**Figure 1:**
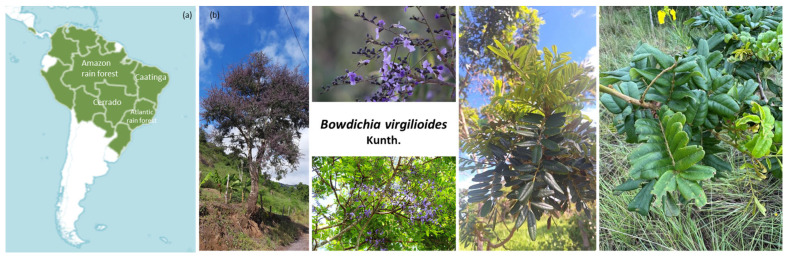
(**a**) Distribution of *Bowdichia virgilioides* in South America (https://powo.science.kew.org/taxon/urn:lsid:ipni.org:names:482144-1, accessed on 30 November 2025) and (**b**) illustrations of the tree, flowers and leaves (https://www.gbif.org/occurrence/gallery?taxon_key=2975476, accessed on 30 November 2025).

The South American (sub)tropical rainforest is characterized by a high diversity and abundance of trees and vines, including a large number of plants belonging to the families Sapotaceae, Lauraceae, Leguminosae (Fabaceae), Melastomataceae and Palmae (Arecaceae). This is the case in particular for the Amazon rainforest, which is rapidly changing in good part due to deforestation, droughts, and fires [1,2]. Humans have largely contributed to the savannization of the Amazon tropical forest, which corresponds to the fragmentation, reduction and alteration of its biodiversity [3,4]. Programs have been set up to promote the reforestation of degraded areas, but it takes time and major resources to limit ecological damages [5,6]. Nevertheless, there are promising tree planting and forest restoration initiatives across LatAm countries, together with international initiatives to restore global forests [7,8]. For example, an ambitious program is ongoing to regrow 73 million trees in the Brazilian Amazon [9]. Reforestation on rural properties combined with sustainable agricultural practices are encouraged [10]. Forest restoration is often performed using native tree species, such as Jeriva palms (*Syagrus romanzoffiana*) and conifers, but other species are also considered, notably nitrogen-fixing Fabaceae species [11,12]. In this context, the plant *Bowdichia virgilioides* Kunth is one of the species selected for reforestation programs in Brazil, notably in the Cerrado and Atlantic coast (Figure 1). An analysis of the plant’s ecology, pharmacognosy and phytochemistry is proposed here. The analysis of the scientific literature pertaining to the plant was performed following the PRISMA 2020 methodology, based on publications (mostly in English) from the databases PubMed, Scopus, and Web of Science (>200 initial records; 125 studies included). The purpose of this systematic review is to provide a comprehensive analysis of the plant’s phytochemistry and pharmacology, to assess the efficacy of the most promising natural products isolated from the plants, and to raise ideas for future studies with this little-known botanical species.

## 2. *Bowdichia virgilioides* Kunth

### 2.1. Distribution, Preservation, and Characteristics

The conservation priority index (CPI) of plant species and habitats points out the areas of the greatest conservation importance for maintaining healthy forests. The CPI is a cumulative value of six attributes *viz.*, altitudinal range, habitat specificities, use values, population size, nativity and endemism, and extraction trend following. The tool can be used for assessing endangered species and to measure impacts on medicinal plant populations, notably for forests of northeastern Brazil [13,14]. A recent CPI analysis of plants species from Northeastern Brazil identified 16 species with a high CPI score (high priority for conservation) and *B. virgilioides* was one of them [15,16]. This tree species, which tends to tolerate climate changes, is well suited for ecological restoration programs [17]. The woody species is abundant (but overused) and presents a large geographic distribution in the Cerrado (Figure 1). It shows a great ability to disperse seeds over long distances. It seems well adapted in a scenario of rapid climate change [18,19].

*Bowdichia virgilioides* Kunth, commonly known as sucupira-preta or black sucupira (or Paricarana tree), is considered as a nucleator and a facilitator in the colonization of open areas by new individuals in Cerrado [20,21]. The plant has been considered in danger of extinction in some parts of Brazil, mainly because of the deforestation and its excessive use in civil construction and furnishing [22]. *B. virgilioides* is highly sensitive to herbicides like glyphosate and paraquat, even at low doses [23,24,25]. Moreover, the overextraction of the plant, associated with biological characteristics such as low density and dormancy of its seeds, contribute to its endangered status [26,27]. This tree, considered important for many birds (notably hummingbirds) and insects (butterfly and wasp species), should be further protected [28,29,30].

### 2.2. Exploitation

*B. virgilioides* can be found in different areas of Brazil and occasionally in urban parks (e.g., the urban park of Recife, Pernambuco state) [31,32]. The fruits of this species sucupira-preta (*B. virgilioides*) shall not be confused with those of sucupira-branca (*Pterodon pubescens*) which is also a medium-sized tree found in the tropical rainforests of South America [33,34]. *B. virgilioides* is adapted to dry and poor soils (xerophytic), and to an intense sun exposure (heliophytic). The trees produce a high-density wood with a long natural durability which is particularly appreciated in home construction and flooring, naval construction and furniture manufacturing [35,36,37,38]. The quality of its wood, very hard and resistant to abrasion, is well recognized [36,39]. With its high wood density and long fibers, *B. virgilioides* is also excellent for the production of a high-quality charcoal [40,41].

The trees are high (8–16 m tall) and moderately thick (trunk 30–50 cm in diameter). The leaves present pubescent leaflets. It is an ornamental tree appreciated for its purple flowers in terminal panicles (florescence in September–October). The inedible fruits are small, flat, with indehiscent pods; they not open when ripe (from October to December) [42]. The tree has been essentially exploited for its wood, notably largely exported to Europe (Portugal) in the 18–19th centuries [43].

### 2.3. Seeds and Plant Propagation

The seeds are thick and highly resistant, and they can be stored for a long period at a wide range of temperatures and are easily propagated in plant nurseries [42,44]. Various methods and recipes have been proposed to overcome the dormancy of sucupira seeds by mechanical or thermal scarification or by immersion in boiling water (100 °C for 10 s), or in a sulfuric acid solution (98% for 10 min) (chemical scarification), in order to facilitate germination. One of the most recent methods, proposed to overcome seed dormancy, consisted of using 70% alcohol combined with a brief (60 s) direct fire [45] (Table 1). Different quality control assays, notably using an electrical conductivity test or an X-ray analysis, have been proposed to evaluate the physiological quality of seeds and their germinative potential [46,47,48]. In fact, dormancy in *B. virgilioides* seeds depends on the degree of the fruits’ maturity. It occurs after the seventh week of floral anthesis when the fruits are predominantly black, dry and brittle [49]. *B. virgilioides* seeds from forests (over seeds from isolated trees in pastures) are recommended for the production of seedlings and the commercialization of seeds for forest restoration [50]. The process for seedlings’ development has been optimized, notably via their cultivation in black soil supplemented with carbonized rice hulls [51]. The application of a phosphorus fertilizer (superphosphate) can also influence the extent of mycorrhizal colonization of roots by arbuscular fungi [52]. *B. virgilioides* is considered relatively tolerant to excess iron [53].

**Figure 2:**
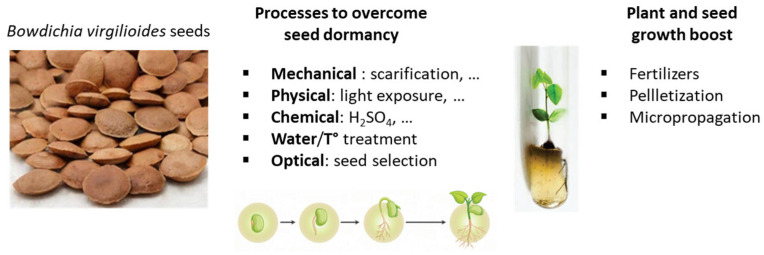
Seeds of *B. virgilioides* and processes to limit dormancy.

The seeds of *B. virgilioides* have been considered rather tolerant to desiccation (orthodox seeds), although a more recent study underlined the rapid loss of desiccation tolerance at the beginning of the germination process [69,70]. Seeds can be stored away from their natural environment. However, the reproduction of this species presents a low efficiency (many seeds do not germinate due to numbness) [71]. It is often useful to boost germination using fertilizers and fertilized tailings. The pelletization of *B. virgilioides* seeds with microalgae, notably *Chlorella sorokiniana* biomass, is an option to prepare seed cement with a high germination capability (Figure 2) [72].

A micropropagation method through the axillary buds of *B. virgilioides* has been proposed [73]. In vitro cultivation processes are being developed, notably using a specific medium (wood plat medium) replaced or combined with biosolids (urban wastes) [74,75]. In vitro plants presenting an embryo-like structure and with vegetative propagules present a reduced development capacity compared to those lacking these morphogenetic structures [76]. The floral ontogeny of *B. virgilioides* is actively studied, notably to better characterize the floral secretory regions and to better comprehend its morphological development [77]. *B. virgilioides* is a valuable plant well studied for its botanical properties, its manufacturing utility, and its associated economic and ecological impacts. The plant’s pharmacology and phytochemistry are also investigated, as discussed in the subsequent sections.

## 3. Pharmacology of *Bowdichia virgilioides* Extracts

*B. virgilioides* is used in traditional medicine against a variety of diseases and health conditions, such as upper airway infections (pharyngitis and bronchitis), healing wounds, diabetes, arthritis and back pain [78,79,80,81]. The plant has been investigated for the treatment of gynecologic affections, including ovarian pain, uterine and vaginal inflammation, and menstrual cramps [82,83]. All the plant’s parts, from roots to leaves, have been used to prepare organic and aqueous extracts subsequently tested against different experimental models. Table 2 summarizes the pharmacological studies performed with *B. virgilioides* extracts and the main observations.

Many studies have been performed with extracts prepared from the bark of *B. virgilioides*. Aqueous bark extracts have been found to display antimicrobial, antiparasitic, anti-inflammatory, antioxidant, antiallergic, antinociceptive, anxiolytic and immune-modulatory effects (Table 2). The antimicrobial potency of the extracts is often limited (with effects observed at doses > 500 µg/mL). Antimalarial activities, also observed when using an ethanolic extract, were noticeable but relatively modest compared to what can be achieved with other medicinal plants used in Latin America (e.g., *Cinchona* and *Artemisia* species). Nevertheless, *B. virgilioides* is one of the many Fabaceae used as antimalarials [84]. The plant contains alkaloids potently active against malaria, such as the quinolizidine alkaloid ormosanine and its analogs (see below).

**Table 2:** Pharmacological activities of *Bowdichia virgilioides* extracts.

Plant Part	Type of Extract	Main Observations	Refs.
Stem bark	Aqueous	Antinociceptive effect. The extract reduced pain (61% and 74% pain inhibition at 200 and 400 mg/kg, respectively) in a model of abdominal writhing induced by acetic acid.	[85]
Stem bark	Aqueous	Antimicrobial and anti-inflammatory effects. Reduction in the damaged area in a wound infected with *S. aureus* and improvement of the wound contraction. The extract induced type-1 collagen deposition and favorized fibroblast accumulation in the wound area.	[86]
Stem bark	Aqueous	Antibacterial activity, notably against *Staphylococcus aureus*, *S. epidermidis* and *S. saprophyticus*. Modest antimalarial activity against *P. falciparum*.	[87]
Stem bark	Ethanolic	Antimalarial activity. Traditional use by the Tacana community (Bolivian Andes). Activity against both *Plasmodium falciparum* chloroquine resistant (D2) and sensitive strains (F32) (IC_50_ = 1 µg/mL). Toxic in vivo against *P. berghei*.	[88] [89]
Stem bark	Aqueous	Anxiolytic effect observed after a single acute oral treatment with the extract (200–400 mg/kg), without compromising motor activity.	[90]
Stem bark	Aqueous (decoction)	Immune effects: alteration of thymocytes and B-lymphocytes functions. Mice orally treated with the extract showed a decreased thymus weight, a reduction in B lymphocytes proliferation, enhanced IL-10 secretion, and decreased TNF-α production.	[91]
Stem bark	Aqueous	Antiallergic effect. The extract inhibited ovalbumin-induced histamine release in mice tissue. It also reduced the TNFα level in the pleural effluent and inhibited the mRNA expression of cytokines IL-5 and CCL11 in pleural leukocytes after ovalbumin challenge.	[92]
Stem bark	Ethanolic	Antinociceptive effect. Inhibition of carrageenan-induced hind paw edema and reduction in exudate volume upon oral treatment at 1000 mg/kg, associated with a marked reduction in leukocyte accumulation. Potent inhibition of writhing and licking (70–80%).	[93]
Bark	Hydroethanolic	Decrease in oxidative stress. Oral administration of the extract (200 mg/kg) for 21 days reduced lipoperoxidation in the plasma and brain, and increased sulfhydryl levels in the brain and muscles.	[94]
Bark	Hydroethanolic	Antioxidant and antinociceptive effects evidenced in models of orofacial pain induced by formalin, glutamate, or capsaicin.	[95]
Inner bark and leaves	Aqueous	Antinociceptive and anti-inflammatory activities in a model of acetic acid-induced writhing in mice, upon oral treatment with the extracts at 200 and 400 mg/kg.	[96]
Root bark	Methanolic	Antihyperglycemic effect in diabetic rats, attributed to the inhibition of intestinal glucose absorption. The extract (500 mg/kg, oral) reduced hyperglycemia after a glucose overload.	[97]
Roots	Hydroethanolic	Rats orally treated with the extract (200 mg/kg) showed a marked reduction in plasma and gastrocnemius tissue lipid peroxidation and oxidative stress (55–66%).	[98]
Roots	Organic	Modest antioxidant activity and marked toxicity in a brine shrimp lethality assay. Best effect with the methanol extract.	[99]
Heartwood	Cyclohexane	Larvicidal activity. Showed 100% mortality at 50–100 μg/mL against fourth instar larvae of *Aedes aegypti*.	[100]
Leaves	Ethanolic	Antibacterial activity. The extract complexed with β-cyclodextrin showed synergistic effects when combined with gentamicin and ciprofloxacin against *Staphylococcus aureus* and *Pseudomonas aeruginosa*.	[101]
Leaves	Entire fresh leaves, untreated	The leaves induced the ripening of banana by increasing respiration and ascorbic acid production, and reducing chlorophyll and pH.	[102]
Leaves	Essential oil from steam distillation	Antimicrobial activity, notably against fungi *Candida albicans*, *C. guilliermondii*, and *C. stellatoidea*.	[103]
Seeds	Supercritical CO_2_ extraction of essential oil.	Solvent-free extraction to produce extracts containing antioxidant small molecules (essentially fatty acids).	[104]
Seeds	Essential oil from steam distillation or cold pressing	A dentifrice formulation prepared with 1% EO from *B. virgilioides* showed adequate organoleptic and physicochemical properties, in addition to an anti-biofilm activity. However, it revealed a marked abrasiveness.	[105]
Seeds	Essential oil obtained after hydrodistillation	Antimicrobial activity, notably against Gram-positive bacteria (*Bacillus subtilis*, *B. bulgaricus*, *Enterococcus faecalis* and *Staphylococcus aureus*), associated with the presence of sesquiterpenes.	[106]

Several studies have underlined the antinociceptive effects of *B. virgilioides* extracts when using both aqueous and ethanolic extracts tested with in vivo pain models. A link with the antioxidant effect has been proposed but the products at the origin of the antinociceptive action have not been clearly identified at present [95]. Similarly, the substances supporting the antiallergic effect of the aqueous root extract remain to be characterized [92]. In contrast, several antioxidant products have been identified from bark extracts, including the major component lupeol which is a well-known antioxidant and anti-inflammatory agent largely present in the plant (see below). Marked anti-inflammatory and anti-edema activities have been observed with both bark and leaf extracts of *B. virgilioides*, even if the in vivo potency of the extracts was inferior to that observed with the reference drug aspirin [96]. It should be noted that in these experiments the pharmacological effects were observed using high concentrations of the crude extracts (200–400 mg/kg) in animal models. The relevance of the effects of the extracts depends on the bioavailablity of the active compounds, their metabolic stability, and biodistribution.

Alcoholic extracts prepared from the plant roots have shown antihyperglycemic and anti-lipid accumulation effects [97,98]. But methanolic extracts presented signs of toxicity in a brine shrimp lethality assay [99]. The plant extracts appeared to be safe, at least in acute toxicity testing. There is a need to reinforce the evaluation of the subchronic toxicity, as well as the geno- and repro-toxicity of the plant extracts.

A recent study underlined the antibacterial potency of a leaf ethanolic extract against pathogenic bacteria such as *Staphylococcus aureus* (Gram-positive) and *Pseudomonas aeruginosa* (Gram-negative) when the extract was complexed with β-cyclodextrin and the resulting encapsulated product combined with the antibiotics gentamicin and ciprofloxacin. This option looks promising with regard to the design new drugs active against multidrug-resistant bacteria, which represents a major clinical need [101]. The leaves and the seeds of *B. virgilioides* have been explored as antioxidant extracts. Noticeably, the seeds have been used to prepare an antimicrobial essential oil, which was then incorporated into a dentifrice formulation for its antibacterial and antibiofilm properties, and aroma [105]. The potential usages of *B. virgilioides* are multiple and diversified, including (i) the use of black sucupira seed oil as a growth-promoting additive for beef cattle [107] and (ii) the ecological exploitation of the untreated fresh leaves themselves to promote the ripening of banana [102]. These two last examples illustrate well the diversity of usages of the plant, beyond its coveted wood.

## 4. Phytochemical Constituents of *B. virgilioides*

Phytochemical studies on *B. virgilioides* can be traced back to the XIX century, with the identification of a “neutral crystalline principle” from the bark of the plant designated alcornoco-rinde (or alcornoque) [108,109]. A phytosterol-like alcohol called alcornine was mentioned but its structure was never established [110]. It corresponded probably to the lupane-type pentacyclic triterpenoid lupeol (**1**), which was isolated much later (1983) from the bark of *B. virgilioides* [111]. This natural triterpene contributed to the anti-inflammatory effects observed when using an ethanolic extract of the stem bark [84]. Lupeol, which can be found in many medicinal plants, displays anti-inflammatory, antioxidant, anticancer, and antibacterial effects, but its poor water solubility and bioavailability strongly limit its pharmaceutical use [112,113,114]. Specific formulations (e.g., PEGylated liposomes and PLGA) with improved biopharmaceutical profiles are being developed [115,116].

**Figure 3:**
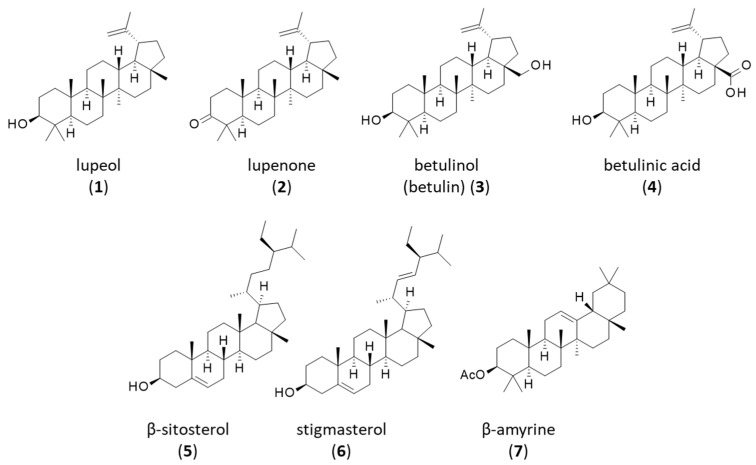
Structure of triterpenes **1–7**.

The related pentacyclic triterpenes lupenone (**2**), betulinol (**3**) (also called betulin), betulinic acid (**4**), β-sitosterol (**5**), stigmasterol (**6**) and β-amyrine (**7**) have been isolated subsequently from the plant’s stem bark or leaves [117,118] (Figure 3). Over the past 40 years, many other natural products have been isolated from *B. virgilioides*, notably alkaloids, terpenoids, and flavonoids, discussed in turn hereafter. The identification of these compounds actually began in the early 1980s with the advent of robust analytical methods (e.g., NMR and LC-MS) specifically applied to natural products’ discovery.

### 4.1. Alkaloids

**Figure 4:**
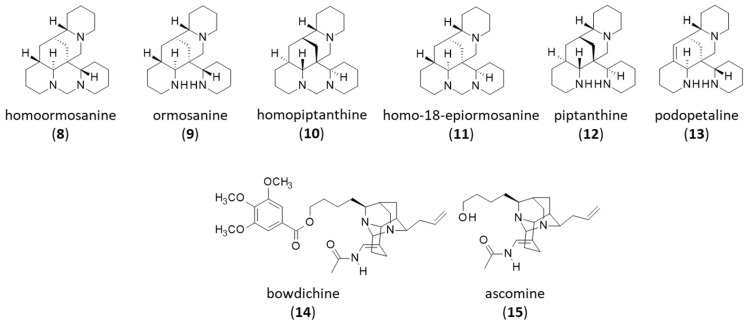
Structure of alkaloids **8–15**.

The first alkaloid isolated from *B. virgilioides* was the quinolizidine alkaloid (−)-homoormosanine (**8**), found in the bark of the plant. Its chemical structure was established by ^1^H-NMR and validated upon comparison with the same product obtained upon the chemical transformation of (+/−)-ormosanine into (−)-homoormosanine in the presence of aqueous formaldehyde [110]. Ormosanine (**9**), found in the stem bark of *B. virgilioides* [119,120], is a potent antioxidant and anti-inflammatory agent. It has been shown to protect neurons in spinal cord-injured rats by regulating the peroxynitrite/calpain activity [121] and to reduce ethanol-induced liver inflammation [122]. Two homoormosanine-type alkaloids homopiptanthine (**10**) and homo-18-epiormosanine (**11**) have been isolated from the stem bark of a Colombian sample of *B. virgilioides* [123]. This series of alkaloids includes piptanthine (**12**) and podopetaline (**13**), also encountered in *B. virgilioides* [117,120,124] (Figure 4). These quinolizidine alkaloids can be isolated from a few other plants (e.g., *Connarus paniculatus*) [125] or obtained via total stereoselective synthesis, as reported from ormosanine and piptanthine [126,127]. Their pharmacological properties have been investigated little. However, homoormosanine (**8**) and ormosanine (**9**) have shown antimalarial activity with a capacity to inhibit the growth of a chloroquine-resistant strain of *Plasmodium falciparum* in vitro (43 and 89% inhibition at 20 µg/mL, respectively) [120].

Two closely related alkaloids with a rare diaza-adamantane skeleton were subsequently isolated from the stem bark of *B. virgilioides*, bowdichine (**16**) and ascomine (**17**) [119]. Ascomine has been initially isolated from the plant *Acosmium panamense*, together with the related diaza-adamantane alkaloids dasycarpumine, panacosmine, and lupanacosmine [128,129]. The pharmacological properties of these two natural products remain unknown at present but it is worth underlining that a few related compounds with a similar diaza-adamantane scaffold were found recently to potentially inhibit mitogen-activated protein kinase (MAPK), based on a computational molecular dynamic analysis [130]. By analogy, it would be interesting to investigate the MAPK binding capacity of compounds (**14**,**15**). Synthetic procedures have been developed to access 1,3-diazaadamantane derivatives [131]. They should facilitate access to derivatives and their pharmacological study.

### 4.2. Di- and Triterpenoids

**Figure 5:**
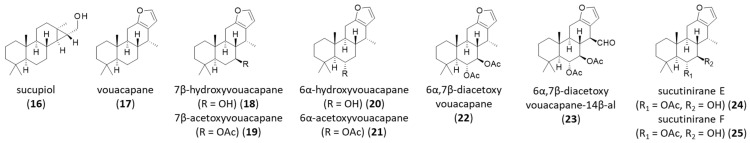
Structure of diterpenoids **16–25**.

In addition to the aforementioned pentacyclic triterpenoids (**1–7**), other diverse terpenoids have been isolated from *B. virgilioides*, notably the pimarane-type diterpenoid sucupiol (**16**) isolated from the seeds of the plant, together with a series of furanocassane-type diterpenoids: vouacapane (**17**), 7β-hydroxyvouacapane (**18**), 7β-acetoxyvouacapane (**19**), 6α-hydroxyvouacapane (**20**), 6α-acetoxyvouacapane (**21**), 6α,7β-diacetoxyvouacapane (**22**), 6α,7β-diacetoxyvouacapane-14β-al (**23**), and sucutiniranes E-F (**24**,**25**) [132] (Figure 5). Sucupiol (**16**) has been structurally characterized but no associated pharmacological activity has been reported thus far. Among tetracyclic diterpenes with a vouacapane skeleton, some compounds were shown to display analgesic and anti-inflammatory properties, notably vouacapane itself [34]. Naturally occurring vouacapane derivatives have shown insecticidal (e.g., 6α-acetoxyvouacapane (**21**)), anti-inflammatory, and cytotoxic (e.g., 6α-acetoxy-7β-hydroxyvouacapane) activities [133,134]. The insecticidal effect of these vouacapanes is consistent with the reported capacity of a cyclohexane extract of *B. virgilioides* to act as a repellent against the neotropical termites *Nasutitermes corniger* [135]. Vouacapanes represent an interesting series of bioactive phytochemicals but more work is needed to better characterize their activity/safety ratio [136]. The design of semi-synthetic vouacapane derivatives is an emerging field aiming to create bioactive pseudo-natural products [137].

**Figure 6:**
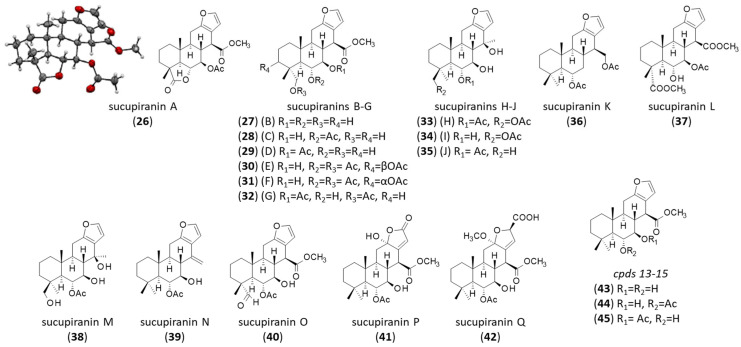
Structure of terpenoids **26–45**.

A series of 17 furanocassane-type diterpenoids has been isolated specifically from *B. virgilioides*. The products, designated sucupiranins A-to-Q (**26–42**), were described by Ohsaki and coworkers [138] (Figure 6). Modest antimalarial activity against *Plasmodium falciparum* K1 was reported in relation to some compounds (notably sucupiranins J and K), and sucupiranins J and P were found to weakly inhibit LPS-induced NO production (IC_50_ = 30.6 and 44.0 μM, respectively) [139,140]. Three more unnamed products (**43–45**) (numbered *13–15* in the original work and initially discovered from a *Pterodon* species [141]) were identified together with sucupiranins A–L [139]. These products deserve further studies considering that related furanocassane diterpenoids have shown interesting anticancer properties [142]. The sucupiranins bear a structural analogy with the cassane-type diterpenes sucutiniranes A-F isolated from the related species *B. nitida* Spruce ex Benth. [143,144]. A semi-synthetic furan-oxidized derivative (cassane butenolide) of sucutinirane F has shown antioxidant and proapoptotic properties [145], thus reinforcing the potential interest of this type of product.

### 4.3. Isoflavones and Other Flavonoids

**Figure 7:**
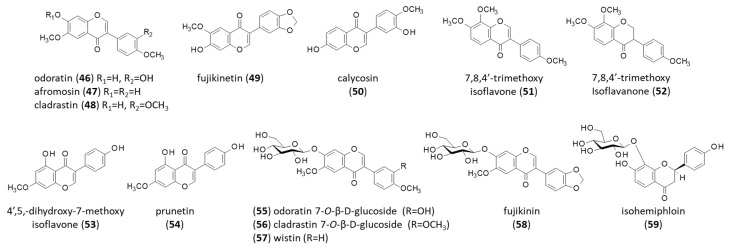
Structure of isoflavones **46–59**.

One of the first isoflavones isolated from *B. virgilioides* was odoratin (**46**) (7,3′-dihydroxy-6,4′-dimethoxyisoflavone), initially isolated from the medicinal Asteraceae *Chromolaena odorata* [146]. This should not be confused with a sesquiterpene lactone with the same name found in *Eupatorium odoratum* and other species. The odoratin isoflavone has been isolated from the roots of *B. virgilioides* together with the related natural products afromosin (**47**), cladrastin (**48**), and fujikinetin (**49**) [147]. These products complement the isoflavone series initially isolated from the plant: calycosin (**50**), 7,8,4′-trimethoxyisoflavone (**51**), and 7,8,4′-trimethoxyisoflavanone (**52**) [148]. More recently, three more isoflavones have been isolated: 4′,5-dihydroxy-7′-methoxyisoflavone (**53**), 3′,7-dihydroxy-4′-methoxyisoflavone (which is calycosin, **50**), and 4′,5-dihydroxy-7′-methoxyisoflavone (also known as prunetin) (**54**) (Figure 7). They revealed a modest potency in terms of inhibiting cathepsins K, L and V in vitro, inferior to that of 8-methoxycoumestrol (discussed below) [149].

The plant also contains glycosylated isoflavones, notably odoratin 7-*O*-β-D-glucoside (**55**) [150], cladrastin 7-*O*-β-D-glucoside (**56**), wistin (**57**), and fujikinin (**58**). Another glucoside, the flavanone derivative isohemiphloin (**59**), was isolated at the same time from the plant [147]. Wistin (4′,6-dimethoxyisoflavone-7-O-β-D-glucopyranoside) is an interesting phytochemical known for its anti-inflammatory effects. It has been shown to reduce the production of nitric oxide (NO) and intracellular reactive oxygen species (ROS) in lipopolysaccharide-stimulated RAW 264.7 macrophages, and to reduce the expression of cytokines (interleukins IL-1β and IL-6) and the pro-inflammatory enzymes iNOS (inducible nitric oxide synthase) and COX-2 (cyclooxygenase-2) [151]. Wistin (**57**) is also an activator of PPARα/γ (peroxisome proliferator-activated receptors α and γ) [152,153] and a modest anticancer agent capable of reducing the invasion and migration of melanoma cells [154]. Initially, the natural product was isolated from the liana *Wistaria floribunda*, and then from many other plants. The compound can be prepared synthetically, starting from the isoflavone afromosine, and glycosylation in the presence of α-acetobromoglucose [155]. Afromosine can be extracted from plants (notably from *Afromosia elata*) or prepared synthetically from deoxybenzoin [156].

Odoratin 7-*O*-β-D-glucoside (**55**), isolated from *B. virgilioides* roots, has been shown to inhibit B- and T-lymphocytes’ activation in vitro. Indeed, it inhibited both T-mitogen (concanavalin A-induced) and B-mitogen (lipopolysaccharide-induced)-stimulated lymphocyte proliferation, whereas the aglycone odoratin (obtained upon the acid hydrolysis of **55**) essentially inhibited T-cell proliferation [150]. Glycoside (**55**) exhibited modest cytotoxicity toward cultured cancer cells, such as HT-29 and CHT-115 human colon cancer cells [100].

**Figure 8:**
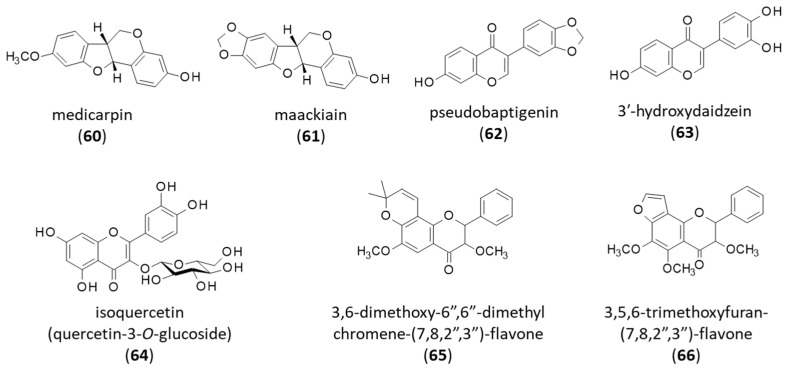
Structure of flavonoids **60–66**.

Medicarpin (**60**) is an isoflavone derivative belonging to the pterocarpan class (Figure 8). It can be found in numerous Leguminosae, including *B. virgilioides*. The tetracyclic product (**60**) and its pentacyclic analog maackiain (**61**) were identified in a cyclohexane extract of the heartwood of *B. virgilioides* [157]. The plant extract revealed a marked larvicidal activity against the fourth instar larvae of the mosquito *Aedes aegypti*, which is a spreading vector for different diseases such as dengue and yellow fever. The extract was shown to contain the two isoflavone derivatives which contributed importantly to the larvicidal activity (LC_50_ = 17.5 and 21.95 μg/mL for medicarpin and maackiain, respectively) [157]. Medicarpin (**60**) is a well-known antioxidant and antiparasitic agent, notably active against the malaria pathogen *Plasmodium falciparum* (IC_50_ = 0.45 µg/m, against *P. falciparum* strain 3D7) [158]. The compound also displays anticancer properties, notably against lung and brain tumors [159,160]. Maackiain (**61**) is an interesting natural product with numerous pharmacological properties (anti-inflammatory, anti-osteolytic, anticancer, antifungal, antimalarial, etc.) [161]. Medicarpin (**60**) is generally more active than maackiain (**61**), at least against fungi.

From the powdered root bark of *B. virgilioides*, Kauffmann and coworkers isolated five compounds: pseudobaptigenin (**62**), 3′-hydroxydaidzein (**63**), quercetin-3-*O*-glucopyranoside (**64**), and 2,4′-dihydroxy-4-methoxybenzophenone and L-(+)-bornesitol (both discussed below) [162] (Figure 8). Pseudobaptigenin (**62**) (also known as psi-baptigenin) is an isoflavonoid acting as an activator of both PPAR-γ mRNA and protein expression, and a PPAR-γ agonist. The compound is presumed to bind to the ligand-binding domain of PPAR-γ [163] but it can target other receptors, for example, estrogen receptor (ER), progesterone receptor (PR), and human epidermal growth factor receptor 2 (HER2), as predicted by a recent in silico analysis [164]. (Iso)Flavonoids often display a limited target selectivity.

3′-Hydroxydaidzein (**63**) is a useful natural product to combat obesity. This daidzein metabolite has been shown to reduce lipid accumulation and to ameliorate high-fat diet (HFD)-induced obesity in mice by stimulating the browning of the white adipose tissue and modulating gut microbiota [165]. The product can be found in diverse plants and in fermented soybean products, such as miso and dou-chi, acting as an antioxidant [166,167]. 3′-Hydroxydaidzein (**63**) has shown marked anti-trypanosomal activity against the parasites *T. brucei rhodesiense* and *T. cruzi* (IC_50_ = 1.62 and 4.7 µg/mL, respectively).

Quercetin-3-*O*-glucopyranoside (**64**), also known as isoquercetin or hirsutrin, is a relatively common natural product found in many plants and considered for the treatment of diverse pathologies, such diabetic retinopathy, osteoarthritis and other inflammatory diseases [168,169]. Isoquercetin is a modulator of various signaling pathways at the nuclear and mitochondrial levels, which is of potential interest to combat neurodegenerative diseases [170,171]. The product was tested in patients with chronic kidney disease (CKD), in combination with sodium nitrite, to improve flow-mediated vasodilation but no major effect was observed [172]. It remains an exploratory research product, largely studied and readily accessible. Isoquercetin (**64**) can be extracted from plant or (bio)synthesized from quercetin and D-allulose [173].

At this stage, we can also mention the two flavonoids 3,6-dimethoxy-6″,6″-dimethylchromene-(7,8,2″,3″)-flavone (**65**) and 3,5,6-trimethoxyfuran-(7,8,2″,3″)-flavone (**66**), isolated from the fresh roots of *B. virgilioides* [174] (Figure 8). The former compound is a rare chromenoflavone found also in the Brazilian species *Diplotropis ferruginea* Benth with its prenylated analog diploflavone, and in the twigs of *Millettia pubinervis* [175]. No major biological activity has been reported for these two products.

### 4.4. Phenols, and Other Compounds

**Figure 9:**
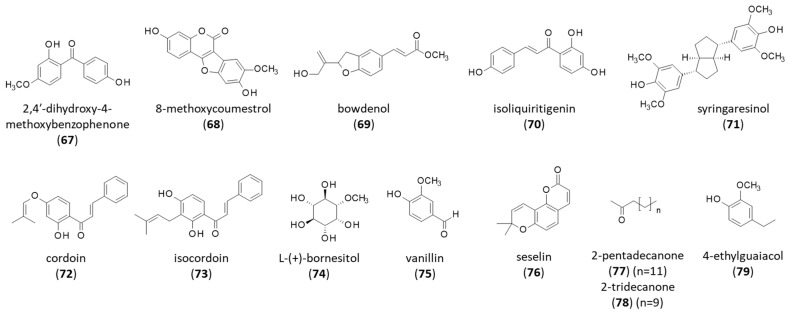
Structure of compounds **67–79**.

The aforementioned product 2,4′-dihydroxy-4-methoxybenzophenone (**67**) is a relatively rare natural product found in a few plants (e.g., *Tolpis* species and *Anemarrhenae* rhizoma) [176,177]. In parallel, this product and close analogs have been used in sunscreen products as ultraviolet absorbers [178,179]. A compound of interest isolated from *B. virgilioides* is 8-methoxycoumestrol (**68**), isolated together with the dihydrobenzylfurane derivative bowdenol (**69**) (Figure 9). The coumestan derivative (**68**) was shown to inhibit the three cathepsin proteases K, L, and V, with a stronger activity toward the latter enzyme (IC_50_ = 17.4 µM for Cat-V) [149]. Cathepsin V, also known as cathepsin L2, is a lysosomal endopeptidase implicated in cancers and vascular disorders [180]. Inhibitors of cathepsin V are being searched for to combat cancer and immunosuppression [181]. 8-Methoxycoumestrol (**68**) could be used as a template to guide the design of novel inhibitors. However, the compound displays other activities, notably a capacity to inhibit Na,K-ATPase pump (isoform α1β1) with an efficacy superior to that of the cardiac glycoside digitoxin (IC_50_: 90 and 287 nM, respectively). Apparently, 8-methoxycoumestrol functions via a distinct mechanism because, unlike digitoxin, it showed no increase in cardiac contractility [182]. Analogs have been designed, such as the synthetic derivative LQB93 (8-methoxy-3,9-dihydroxy coumestan), to act as viper snake (Bothrops) venom enzyme inhibitors [183,184].

The 2,3-dihydrobenzofuran derivative bowdenol (**69**) was first isolated from the bark of *B. virgilioides* together with the triterpenoids lupeol (**1**) and lupenone (**2**), and the phytosterols β-sitosterol (**5**) and stigmasterol (**6**). The biosynthetic origin of the compound has been associated with the shikimate and aromatic amino acid (e.g., tyrosine) biosynthetic pathways [185]. Bowdenol was later reisolated from the wood of the plant together with isoliquiritigenin (**70**), syringaresinol (**71**), and the aforementioned isoflavones [148].

The two prenylated chalcones cordoin (**72**) and isocordoin (**73**) have been isolated recently from the roots of *B. virgilioides*, together with lupeol, lupenone and stigmasterol [99]. Cordoin (**72**), also known as derricidin, has been shown to function as a transcriptional repressor of the Wnt/β-catenin pathway, and is of potential interest to tackle colon cancer [186]. It is also an inhibitor of soybean 15-lipoxygenase (IC_50_ = 0.6 µM) [187]. Isocordoin (**73**) displays anti-inflammatory and anticancer effects [188]. The natural product has been exploited for the design of hemisynthetic derivatives endowed with anti-edema, antinociceptive, or anti-oomycete activities [189,190].

L-(+)-bornesitol (**74**) is a known carbohydrate, initially identified in sweet peas (*Lathyrus odoratus* L. cv. Diana) [191] and found in other plants including *B. virgilioides* [136]. This cyclitol has been characterized as an inhibitor of TPA (12-O-tetradecanoyl-13-acetate)-mediated NF-kB activation (IC_50_ = 27.5 µM) [192]. In vivo, compound (**74**) was shown to induce the endothelium- and NO-dependent vasodilatation of rat aorta and to reduce blood pressure [193]. This antihypertensive agent is present in different medicinal herbal preparations, notably those containing the berry-shaped Brazilian fruit mangaba (*Hancornia speciosa*) [194]. Other minor products have been isolated from *B. virgilioides*, such as 4-hydroxy-3-methoxybenzaldehyde, which corresponds to the aroma vanillin (**75**) and is commonly utilized in food, beverages, and cosmetics but is also of interest as an anti-inflammatory agent [195].

Finally, it is worth mentioning volatile compounds isolated from an oil prepared by the steam distillation of a hexane extract from the roots of *B. virgilioides*. Various volatile products were identified, including the pyranocoumarin seselin (**76**) and the more abundant products 2-tridecanone and 2-pentadecanone (**77**,**78**), ethylguaiacol (**79**) [196]. The most abundant product in this root extract was 2-tridecanone (**77**) (54.6% in the root oil), an allelochemical toxic to some insects [197] and occasionally used in fragrance and skin products [198]. Seselin (**76**) is a well-known anti-inflammatory pyranocoumarin targeting kinase Jak2 in macrophages and is of interest for its potential to combat sepsis [199].

## 5. Discussion

The plant *Bowdichia virgilioides* is common in several regions of South America, notably in the savannah of Central Brazil. It is a dominant woody species of the cerrado flora. Trees of various sizes can be found depending on their habitat. Large evergreen trees (up to 36 m tall) are present in the rainforest, whereas smaller trees (8–16 m tall) are usually observed in the savannah. For a long time, these trees are exploited commercially for robust and valuable timber. The high density and resistance of its wood are highly prized in construction. Beyond its wood, the plant has fine blue/purple flowers when in bloom and is appreciated for its ornamental value and for landscaping. Moreover, it is an important species considered for the reforestation of degraded areas and forest regeneration. For these reasons, the plant, its ecological environment, and its properties are well studied. Notably, methods to brake seed dormancy and cultivation processes are continuously investigated. Processes to facilitate large-scale propagation in plant nurseries have been deployed [44].

The ethnomedicinal value of *B. virgilioides* has been known of for a long time. Different communities in Brazilian Amazonia use the plant as a remedy against ulcers, parasitic infections and other ailments. All the parts of the plant are useful. For example, the inner trunk bark, scrapped until a fine powder is obtained, is used as a poultice to improve healing of ulcers. Alternatively, a piece of trunk bark boiled with water provides an oral remedy used to treat dysentery and malaria [88]. As defined in Table 2, all the plant’s parts, from roots to leaves, can be used to combat a range of pathologies, with plant decoctions, essential oils, and organic/hydroalcoholic extracts. It is recommended to prioritize the use of the renewable part of the plant (such as the leaves) to preserve the species in the Cerrado biome.

The medicinal use of *B. virgilioides* resin (oil) has already been quoted about one century ago [200,201] and it remains largely underlined nowadays, notably for the treatment of inflammatory symptoms [80,202]. The seeds provide also an important medicinal material. The infusion of seeds can be used to control body uric acid, tonsillitis, arthritis, asthma, gonorrhea, ovarian and uterus cysts, organic weakness, skin diseases, diabetes, sore throat, spasmodic pain, wounds, bleeding, inflammations, rheumatism, syphilis, and worms. In addition, the seeds’ oil can be used in the treatment of arthritis and joint pains [41]. The recent mention of the incorporation of an essential oil (EO) of *B. virgilioides* in toothpaste to reinforce its antibacterial and antibiofilm activities illustrates well the diversity of usages of the plant [105]. Various types of *B. virgilioides*-containing products are being explored, either in the form of EOs or as ethanolic leaf extract complexed with β-cyclodextrin to combat bacterial infections [101]. The multiplicity of biological effects reported with *B. virgilioides*-containing preparations encourages further studies on this good-for-everyone plant. The medicinal usages of the plant are often supported with in vitro data but solid proofs of efficacy in animal models are very limited. More in vivo data are needed. In particular, there is a need for experimental models that directly validate popular indications, such as articular inflammation assays or tissue repair models. Thus far, experimental studies have addressed primarily the antioxidant and antimicrobial properties of the plant extracts, whereas the anti-inflammatory activities have been less investigated. The plant is commonly used to treat rheumatism and wound healing. Pharmacological studies are needed to support these major traditional usages of the plant.

**Figure 10:**
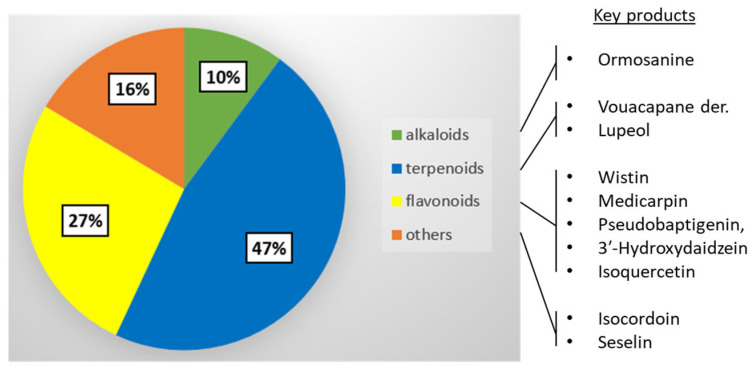
Categories of natural products isolated from *B. virgilioides*. Terpenoids (37), alkaloids (8), flavonoids (21), and other products (13), for a total of 79 products (number of products identified in each subgroup). Examples of pharmacologically important products are indicated.

The wide variety of bioactive compounds present in extracts from *B. virgilioides* has been previously underlined, with the mention of many product types (anthocyanins, alkaloids, terpenoids, polyphenols, saponins and steroids) but without specific details [91]. The present phytochemical analysis provides an updated view of the bioactive natural products isolated thus far with the plant, with about 80 products belonging essentially to four chemical families: terpenoids, alkaloids, flavonoids, and other products (Figure 10). The molecular diversity is not surprising for this type of plant in the Fabaceae family, which is notoriously rich in bioactive phytochemicals [203]. In fact, the diversity of small molecules isolated from *B. virgilioides* not only reflects the intrinsic richness of the plant but also the number and extent of phytochemical studies dedicated to this species. Overall, Fabaceae species (legumes) are largely investigated, and more so when it concerns a species of medicinal, ecological, agronomic and economic interest as in the present case. Fabaceae represent the second largest family of medicinal plants (751 genera and >19,500 plant species), many of which are or have been used as traditional medicines, notably to treat microbial infections [204,205]. The range of phytochemicals found in *B. virgilioides* is reminiscent to that found in other Fabaceae, with a rich terpenoid profile in particular [206,207].

Among the many natural products isolated from *B. virgilioides*, one can distinguish those which have been chemically characterized but not deeply investigated (e.g., sucupiol and fujikinin), from those with defined pharmacological properties, such as lupeol, for example. The two typical *Bowdichia* products bowdichine (alkaloid) and bowdenol (dihydrobenzofurane) are emblematic of the plant but are little known, with no associated mechanism or privileged target. Beyond their identity, they have accrued little biological interest thus far. In sharp contrast, a dozen compounds seem to be essential and directly implicated in the multiple activities observed with plant extracts (Figure 10).

In the alkaloid series, the most important product is arguably ormosanine, endowed with potent antimalaria activity [120]. This compound is also known for its antioxidant and anti-inflammatory properties [122]. In the terpenoid series, there are little-known compounds (e.g., sucupiol), and better-known compounds characterized by anti-inflammatory effects, such as vouacapane derivatives [132]. Vouacapane diterpenoids are interesting bioactive entities but they often present poor solubility in aqueous media and a limited bioavailability. There are options to improve these difficulties via the design of functionalized derivatives and the construction of pseudo-natural product libraries, as reported recently [136], or via the encapsulation of the product into micro/nano-particles to improve delivery with adapted formulations [208]. However, in the terpenoid group, the main product of interest is lupeol, for which innovative strategies to improve its bioavailability have already been deployed with the objective of enhancing its clinical efficacy [114,209,210]. It is a somewhat “a jack of all trades”, multifaceted product, present in a hundred plants [114], even considered a “magical drug” in some cases [211], but it remains a polyfunctional experimental product not an approved drug (a jack of all trades, master of none, in other words). The sucupiranins provide a large series of terpenoids but the pharmacological knowledge of these products remains excessively limited at present.

Five bioactive products from *B. virgilioides* dersevre mention (i) the anti-inflammatory agent wistin (isofavone) acting as regulator for pro-inflammatory enzymes and PPARα agonist, (ii) the antioxidant and insecticidal product medicarpin (a pterocarpan), (iii) the anti-inflammatory benzopyranone pseudobaptigenin, (iv) isoflavone glycoside 3′-hydroxydaidzein (isofavone), and (v) isoquercetin, well-known as an antioxidant and neuroprotective agent. Interestingly, all five isoflavones have exhibited anticancer properties, against melanoma cells for wistin [154], lung and bladder cancer cells and glioblastoma for medicarpin [159], breast cancer cells for pseudobaptigenin [164], skin, nasopharyngeal, and lung cancers for isoquercetin [212,213]. If the supportive experimental data are limited for the first four products, they are particularly robust for isoquercetin (isoquercitrin), which is now being considered for clinical translation in oncology [214]. The product, evaluated in patients with sickle cell disease, has shown a favorable safety profile [215]. It is a useful product to regulate tumor cell proliferation, acting directly on cancer cells and indirectly via the immune system [216]. This compound emerges as a pivotal bioactive constituent of *B. virgilioides* extracts, of interest to combat cancers but also inflammatory diseases like sickle cell disease and chronic kidney disease [172,215].

The last two compounds of interest are the prenylated chalcone isocordoin and the pyranocoumarin seselin. The first is an antiparasitic agent, active against *Leishmania mexicana* promastigotes and *Trypanosoma cruzi* epimastigotes (IC_50_ = 7.70 and 7.00 µM, respectively), but the compound presents cytotoxicity in the same dose range [217]. Interestingly, the replacement of the two phenol-OH groups with methoxy groups reinforced the antitrypanosomal activity while considerably minimizing the cytotoxic action (IC_50_ = 1.50 and 164.0 µM against *T. cruzi* and MDCK (Madin–Darby canine kidney) cells, respectively) [217]. Isocordoin itself is weakly active but there is room to improve its (antiparasitic, antifungal, and anti-inflammatory) properties through the development of analogs [189,190,218] The second compound, seselin, is an antifungal and antiviral agent. Its activity against the fungus *Botrytis cinerea* that damages crops (e.g., tomatoes and eggplants) has been associated with its capacity to disturb Ca^2+^ homeostasis, leading to fungal cell death [219]. The compound is interesting as it exhibits a marked JAK2-dependent anti-inflammatory activity in parallel with its antifungal action [199]. It is certainly an important contributor to the bioactivity of *B. virgilioides* extracts. Altogether, a dozen bioactive natural products account for the diverse pharmacological activities observed with *B. virgilioides* extracts. Studies shall continue to define the potential contribution of the other products. There are limitations associated with the use of the plant and its bioactive products. Many of the aforementioned phytochemicals have been tested using in vitro systems only and/or via in silico approaches. Proofs of efficacy in in vivo experimental models are limited, as well as the definitions of the molecular targets and the pathways engaged in their bioactivities. Bioavailability and (acute and subchronic) toxicology studies are also needed to better appreciate the therapeutic potential of these compounds, providing that the test compounds can be adequately formulated to offer sufficient exposure in animal studies. In other words, a deeper understanding of the pharmacology of *B. virgilioides* products is needed.

Altogether, the analysis afforded a repertoire of about 80 natural products isolated from *B. virgilioides*, with a large proportion of terpenoids. The most bioactive products were identified, along with their mechanism of action and pharmacological effects. This medicinal plant, rich in bioactive compounds, should be protected and further investigated. A recent analysis by the Brazil Flora Group underlined the extreme diversity of the Leguminosae (Fabaceae) in the country, and pointed out *Bowdichia virgilioides* Kunth as one of the key trees that dominate the *Cerrado* savannah [220]. It is an important species for wood production and reforestation [221], but is also a useful medicinal plant used for a long time to treat diverse human pathologies. All parts of the tree can be used to prepare bioactive extracts. Almost 80 natural products have been identified thus far from *B. virgilioides*, including a dozen key substances with a known medicinal interest. All these data highlight the absolute necessity to protect this endangered species, to develop its cultivation and propagation, and to extend the phytochemical investigations. It is not only a local Cerrado topic but also a general issue of worldwide concern to preserve our living environment.

## Figures and Tables

**Table 1:** Processes used to overcome dormancy of *B. virgilioides* seeds.

Parameters	Main Observations	Ref.
Thermal scarification	Comparison of processes to overcome seeds dormancy. Thermal scarification (water at 80 °C for 10 min) is more efficient than chemical and mechanical scarification (90% germination vs. 70%).	[54]
Seed color	Study of the relation of seed coat color with permeability and viability. Seeds with a reddish color are more permeable and present a greater physiological potential favoring germination.	[55]
Luminosity	Impact of luminosity (light and dark) on germination of sucupira seeds after a mechanical scarification. Germination in the presence of light is largely superior compared to in the absence of light. Impact of shading on plant and seedling growth. Unshaded leaves can fix much more CO_2_ than leaves beneath the grass canopy.	[56] [57]
Chemical treatment and burning	Treatment of colored seeds (red/orange) with 70% alcohol and fire for 60 s to overcome seed dormancy	[45]
Chemical treatments	Comparison of methods for improving seed germination. Immersion in sulfuric acid (10 min) and in hot water (80 °C, 5 min) is recommended for overcoming seed dormancy. The same as above, with or without additional immersion in 2.5% sodium hypochlorite for 1 min. Immersion in sulfuric acid (4–8 min) followed with neutralization with CaCO_3_ (2%, 3 min) to improve germination rate and speed. Evaluation of the impact of osmotic changes (induced with polyethyleneglycol PEG-6000) on seed germination. Water stress hindered seed germination.	[58] [59,60,61] [62] [63]
Water imbibition	Water imbibition (48–36 h) of seeds increases the percentage and speed of germination.	[64]
Water and temperature analyses	Combination of a 30 °C temperature and volume of water (2.5 × weight of the seeds) to favor seed germination.	[65]
Morphometry + chemical treatment	Optimal process to break dormancy: treatment with boiling water (100 °C, 10 sec.) + immersion in hypochlorite (2%, 5 min.) of orange/red seeds.	[46]
Germinability	Analysis of seed germinability parameters: time to germination, speed, frequency, and synchrony of the seed germination.	[66]
Temperature and light	Influence of temperature and light on seed germination. Optimal germination T° = 25 °C; seeds indifferent to light conditions.	[67]
Moisture content	Conditions for analysis of seed water content (103 °C for 17 h or 132 °C for 1 h).	[68]

## Data Availability

Not applicable.

## Abbreviations

The following abbreviations are used in this manuscript:

CPI	Conservation priority index
COX-2	Cyclooxygenase-2
HER2	Epidermal growth factor receptor 2
iNOS	Inducible nitric oxide synthase
MAPK	Mitogen-activated protein kinase
NO	Nitric oxide
PPAR	Peroxisome proliferator-activated receptor
ROS	Reactive oxygen species
